# Metabolic Redox Modulation by *Agaricus bisporus* Aqueous Extract in Honey Bee Cells

**DOI:** 10.3390/molecules31122011

**Published:** 2026-06-09

**Authors:** Đura Nakarada, Uroš Glavinić, Jevrosima Stevanović, Uroš Gašić, Marko Ristanić, Miloš Mojović, Zoran Stanimirović

**Affiliations:** 1 Center for Physical Chemistry of Biological Systems, BioScope Labs, Faculty of Physical Chemistry, University of Belgrade, 11158 Belgrade, Serbia; djura@ffh.bg.ac.rs (Đ.N.); milos@ffh.bg.ac.rs (M.M.); 2 Department of Biology, Faculty of Veterinary Medicine, University of Belgrade, 11000 Belgrade, Serbia; uglavinic@vet.bg.ac.rs (U.G.); mristanic@vet.bg.ac.rs (M.R.); zoran@vet.bg.ac.rs (Z.S.); 3 Department of Plant Physiology, Institute for Biological Research “Siniša Stanković”, National Institute of the Republic of Serbia, University of Belgrade, 11060 Belgrade, Serbia; uros.gasic@ibiss.bg.ac.rs

**Keywords:** button mushroom, *Apis mellifera*, redox modulation, EPR, oxidative stress

## Abstract

The western honey bee (*Apis mellifera*) is increasingly exposed to environmental stressors that affect redox homeostasis, leading to imbalances in cellular functions. Natural bioactive compound-based nutritional strategies show promise in reducing oxidative stress while preserving redox signaling. In this study, we investigated the chemical composition, cytotoxicity, and redox-modulating effects of an aqueous extract of the edible mushroom *Agaricus bisporus* on the AmE-711 honey bee cell line. High-resolution Orbitrap LC–MS analysis revealed a chemically diverse extract comprising polyols, organic acids, amino acids, phosphorylated sugars, nucleotide derivatives, phenolic, and lipid-related compounds. Among the identified metabolites were mannitol, malic acid, citric acid, glutamic acid, and uridine diphosphate N-acetylglucosamine, providing a biochemical basis for potential metabolic and redox-related activity. Cell viability assays demonstrated that *A. bisporus* extract exhibited no significant cytotoxicity under the experimental conditions. Electron paramagnetic resonance (EPR) spectroscopy with the TEMPONE spin probe showed that untreated cells exhibited only minimal signal reduction (4.20%), while treatment with the extract alone caused a moderate decrease (12.08%), indicating the absence of reductive stress. Oxidative stress induced by hydrogen peroxide resulted in a pronounced TEMPONE signal reduction (37.88%), whereas co-treatment with the *A. bisporus* extract substantially attenuated this effect, lowering the signal reduction to 15.34%. These findings suggest that the aqueous *A. bisporus* extract may help preserve basal redox activity while attenuating peroxide-induced oxidative stress in AmE-711 honey bee cells. Rather than acting as a potent radical scavenger, the extract appears to function as a mild redox modulator or stabilizer under the tested conditions, which may be beneficial for honey bee cellular redox balance. These results support further investigation of physiologically appropriate *A. bisporus*-based dietary supplements for mitigating oxidative stress in apicultural systems.

## 1. Introduction

Through its pollination activity, the western honey bee (*Apis mellifera*) is essential to both agricultural productivity and ecosystem stability [1]. However, a number of environmental stressors, such as pesticide exposure, pathogens, nutritional deficiencies, and habitat degradation, have caused significant declines in honey bee populations in recent decades [2]. The disruption of cellular redox homeostasis, leading to oxidative stress that compromises immune competence, metabolic function, and overall bee colony resilience, is a common physiological consequence of these stressors [3,4].

As inevitable byproducts of aerobic metabolism, reactive oxygen species (ROS) play crucial signaling roles in insects, including regulation of immunity, development, and stress responses [5]. Tightly regulated redox signaling is essential for preserving cellular homeostasis in honey bees [6]. However, an excessive build-up of ROS can overpower the natural antioxidant defenses, causing oxidative damage to proteins, lipids, and nucleic acids [7]. On the other hand, excessive ROS suppression by high-dose antioxidant supplementation may cause reductive stress and disrupt physiological signaling pathways [8,9]. Therefore, it is becoming more widely acknowledged that a more physiologically appropriate approach to promoting insect health is to use nutritional interventions that stabilize rather than eradicate redox activity.

Because of their chemical diversity and generally low toxicity, natural products are becoming more and more popular as dietary supplements in apiculture [10,11]. A significant number of natural-based diet supplements were investigated and proclaimed beneficial for honey bee health, such as plant-based bioactive molecules (e.g., polyphenols, amino acids, and secondary metabolites) and essential oil constituents (e.g., monoterpenes, sesquiterpenes, and phenolic derivatives) [10,12,13,14,15,16], but studies on their antioxidant efficacy in honey bees are limited [17,18,19].

The “white button” mushroom *Agaricus bisporus* is one of the most extensively grown edible fungi in the world and is known to contain a wide range of bioactive substances, such as polyols, organic acids, amino acids, polysaccharides, and phenolic derivatives [20,21]. Its remarkable nutritional, medicinal, and cosmetic values are reviewed by Usman et al. [22]. This mushroom is considered a healthy food due to multiple bioactive properties, including antioxidant and immunomodulatory effects [22,23]. The potential of *A. bisporus* in animal nutrition and veterinary medicine is less studied, but available studies indicate favorable effects on production traits and animal health [24,25,26,27,28]. Although *A. bisporus* has been linked to immunomodulatory, anti-inflammatory, and antioxidant effects in mammalian systems [28,29,30,31], little is known about its cellular mechanisms of action and possible advantages in insects. Among insects, investigations of *A. bisporus* extract were conducted only on honey bees and a honey bee cell line, and all showed its beneficial effects. Better survival and reduced levels of *Nosema ceranae* infection [32,33], up-regulation of immune-related genes [32], and mitigation of pesticide-induced oxidative stress were recorded in caged bees supplemented with the *A. bisporus* extract [33]. Furthermore, antigenotoxic/genoprotective effects of the same extract have been revealed by the Comet assay on honey bee cells [34]. Finally, in a field experiment, an extract of *A. brasiliensis* (a relative species of *A. bisporus*) improved the strength of and contributed to the increase in honey and pollen stores in supplemented bee colonies [35].

Recent studies have suggested that, in some mushroom extracts, biological activity may not depend exclusively on high concentrations of phenolic antioxidants, but could also involve the synergistic action of low-molecular-weight metabolites that support metabolic balance and endogenous redox regulation [36,37,38]. Compounds such as mannitol, organic acids involved in the tricarboxylic acid cycle, amino acid derivatives, and nucleotide-related metabolites have been proposed as potential modulators of cellular redox status through effects on metabolism, redox cofactors, and stress-response pathways [39,40].

Although previous studies demonstrated beneficial effects of *A. bisporus* extracts in honey bees, including reduced *Nosema ceranae* infection, improved survival, mitigation of pesticide-induced oxidative stress, and antigenotoxic activity, the underlying cellular redox mechanisms remain insufficiently characterized. In particular, no previous study has combined high-resolution LC–MS metabolite profiling with direct EPR-based monitoring of intracellular redox dynamics in honey bee-derived cells. Furthermore, the effects of *A. bisporus* metabolites on the redox status of the AmE-711 honey bee cell line have not yet been investigated.

The AmE-711 cell line, derived from *A. mellifera* embryonic tissues, represents a well-established in vitro model for investigating cellular responses in honey bees under controlled experimental conditions. Although embryonic cells do not fully reproduce the complexity of adult bee physiology, they retain conserved metabolic and redox-regulatory pathways relevant to oxidative stress responses [41]. Their use enables reproducible assessment of cytotoxicity, oxidative imbalance, and cellular redox modulation while minimizing biological variability inherent to whole-organism studies.

An aqueous *A. bisporus* extract was studied with the aims: (i) to characterize its metabolite profile using high-resolution LC-MS; (ii) to assess its cytotoxicity in AmE-711 honey bee cell line; and (iii) to examine its effects on cellular redox dynamics under basal and oxidative stress conditions using electron paramagnetic resonance (EPR) spectroscopy. By integrating high-resolution qualitative LC–MS metabolite profiling with EPR-based monitoring of intracellular redox activity in AmE-711 cells, this study provides new insights into the potential mechanisms underlying the redox-modulating effects of *A. bisporus* extract.

## 2. Results and Discussion

### 2.1. Chemical Profiling of A. bisporus Extract

There are several groups of biologically active compounds previously found in the aqueous extract of the white button mushroom: water-soluble polysaccharides and phenolic compounds [42]. Vunduk et al. [43] identified 31 compounds identified in hydrophilic extracts of *A. bisporus*, including amino acids, organic acids, saccharides, and nucleic acid derivatives. They contribute to antioxidant, anticancer, antidiabetic, and antimicrobial effects [42,44].

High-resolution Orbitrap LC–MS analysis enabled the detection of numerous metabolites in the aqueous extract of *A. bisporus*. Compound annotations were assigned based on accurate mass measurements, MS/MS fragmentation patterns, and comparison with literature data and databases. Since authentic reference standards were not used, all metabolite identifications should be considered tentative. The analysis revealed the highly diverse chemical profile of the *A. bisporus* aqueous extract. Among the annotated metabolites were polyols, organic acids, amino acids, phosphorylated sugars, nucleotide derivatives, and a number of phenolic and lipid-related compounds (Table 1). The results indicate a broad spectrum of primary metabolites, alongside the presence of secondary metabolites. Most of the annotated constituents were already found in *A. bisporus* [31,45,46,47,48,49,50,51,52,53,54], while eight metabolites tentatively detected in the present extract (tetrose, tetrahydroxypentanoic acid, *N*-(1-Deoxy-D-mannitol-1-yl)-L-glutamic acid, methylcitric acid, 2-furoic acid, phelligridin D, phelligridin C and betulin-3-caffeate) have not, to the best of our knowledge, been previously reported in aqueous extracts of *A. bisporus*.

Hexane-1,2,3,4,5,6-hexol (likely mannitol) was among the constituents. It is a well-known major polyol in mushrooms and plays a significant role in osmotic regulation and oxidative stress protection [55,56]. The presence of mannitol is in accordance with the previous findings describing it as a dominant soluble carbohydrate in *A. bisporus* fruiting bodies [57]. Mannitol possesses hydroxyl radical scavenging properties and therefore represents a plausible contributor to the redox-modulating activity observed in biological assays [58,59].

Organic acids were also a prominent group of compounds in the extract. Malic acid, citric acid, and fumaric acid were tentatively identified, possibly indicating an active tricarboxylic acid (TCA) cycle signature in the mushroom tissue [60,61]. These organic acids affect the cellular redox balance through the mitochondrial metabolism [62,63]. Additionally, methylcitric and aconitic acids were annotated, further supporting the presence of TCA-related intermediates.

Several amino acids and amino acid derivatives were tentatively identified, including aspartic acid, glutamic acid, threonine, and pyroglutamic acid. Glutamic acid is interesting due to its central role in nitrogen metabolism and biosynthesis of glutathione [64]. Pyroglutamic acid has been associated with antioxidant activity and regulation of cellular redox homeostasis [65,66], suggesting a possible mechanistic link to the observed cellular responses.

The presence of phosphorylated sugars and nucleotide-related metabolites was also found in the extract. These include glucose phosphate, glycerol phosphate, *N*-acetylglucosamine phosphate, uridine monophosphate (UMP), and uridine diphosphate *N*-acetylglucosamine (UDP-GlcNAc). This metabolite is a major precursor for glycosylation and cell wall biosynthesis and could be related to the structural complexity of fungal polysaccharides [67,68]. Glycosylation-related metabolites and fungal glycoconjugates have been associated with immunomodulatory and cytoprotective effects through their involvement in cell signaling, immune recognition, and stress-response pathways [69,70,71]. However, since the LC–MS analysis performed here is qualitative and does not provide quantitative concentration data, whether the detected levels of these compounds are sufficient to generate the reported biological effects in honey bee cells cannot be directly assessed from the present data.

Various phenolic and bioactive secondary metabolites were annotated despite the aqueous extraction procedure. Compounds tentatively assigned as phelligridin C and D were detected. These metabolites have previously been associated with antioxidant and anti-inflammatory activities and may represent potential contributors to the observed biological effects [72,73]. A compound tentatively annotated as betulin-3-caffeate was also detected. This metabolite belongs to a class of conjugated triterpenoid–phenolic derivatives known for their antioxidant and antimicrobial properties [74,75].

The presence of lipid-related metabolites was also noted. These include dihydroxyoctadecadienoic acid and long-chain hydroxy fatty acids (hydroxydocosanoic and hydroxytetracosanoic acids). These compounds may affect the membrane integrity and signaling pathways and have been associated with anti-inflammatory and cytoprotective effects [76,77].

It should be emphasized that the LC–MS analysis performed in this study was qualitative and intended primarily for metabolite profiling. Therefore, the relative abundance of individual metabolites was not determined, and the contribution of specific compounds to the observed biological effects cannot be conclusively established. The observed redox-modulating activity likely results from the combined and potentially synergistic action of multiple extract constituents.

The chemical profile of the *A. bisporus* aqueous extract reflects a complex mixture of primary metabolites that support cellular metabolism and redox balance, together with secondary metabolites possessing recognized antioxidant potential. The coexistence of polyols, organic acids, amino acids, nucleotide derivatives, and phenolic compounds [78] could provide a plausible biochemical basis for the low cytotoxicity and redox-modulating effects in honey bee cells, which will be further discussed in the present study. These findings are consistent with previous reports [79,80] highlighting *A. bisporus* as a source of multifunctional bioactive compounds and support its further investigation as a natural agent for mitigating oxidative stress in *Apis mellifera*.

### 2.2. Cell Viability Measurement Results

Viability of AmE-711 cells in the negative control group (90.73 ± 1.07%) and in the group treated with the *A. bisporus* aqueous extract (90.24 ± 1.22%) was more than 90%, while viability of the cells in the positive control group treated with 100 µM H_2_O_2_ remained above 85% (85.81 ± 2.43%). Results are expressed as mean ± SE (*n* = 3). Normality of the data was confirmed by the Shapiro–Wilk test (all *p* > 0.05), and homogeneity of variance was verified by the Levene test (F = 0.75, *p* = 0.513). One-way ANOVA revealed no statistically significant differences in cell viability among the three experimental groups (F(2,6) = 2.58, *p* = 0.155) (Figure S1). This shows that none of the experimental treatment procedures caused significant cytotoxicity. Therefore, the levels of cell viability confirm that the procedures used in the experiment and applied extract concentration were adequate for subsequent analyses in this study.

### 2.3. Redox Modulation in Apis mellifera Cells by A. bisporus Extract

**Figure 1:**
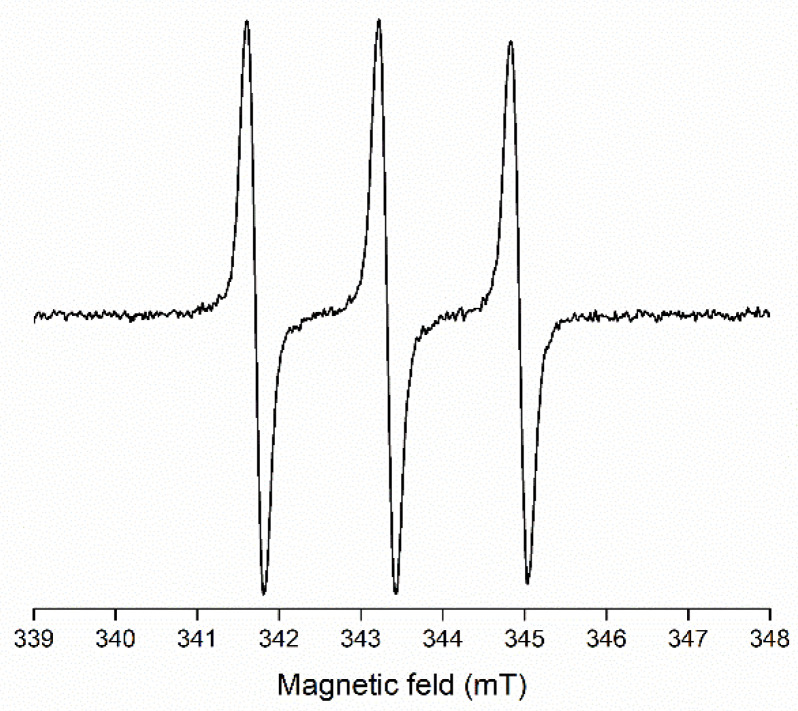
The representative EPR spectrum of TEMPONE.

In this study, the redox activity of *A. mellifera* AmE-711 cells and the modulatory effects of the *A. bisporus* aqueous extract were investigated using EPR spectroscopy in combination with the spin probe TEMPONE (Figure 1). The application of this spin probe has previously enabled the direct observation of intracellular redox changes, providing insight into oxidative stress dynamics and antioxidant interactions in living cells [81,82]. The decrease in TEMPONE EPR signal intensity reflects the reduction of the spin probe to its EPR-silent hydroxylamine form by intracellular reductants—including NADH, gluta-thione, and ascorbate—as well as by the net redox activity of the cellular environment. This approach therefore provides a direct measure of the overall intracellular reducing/redox capacity rather than a measure of a single radical-scavenging reaction.

**Figure 2:**
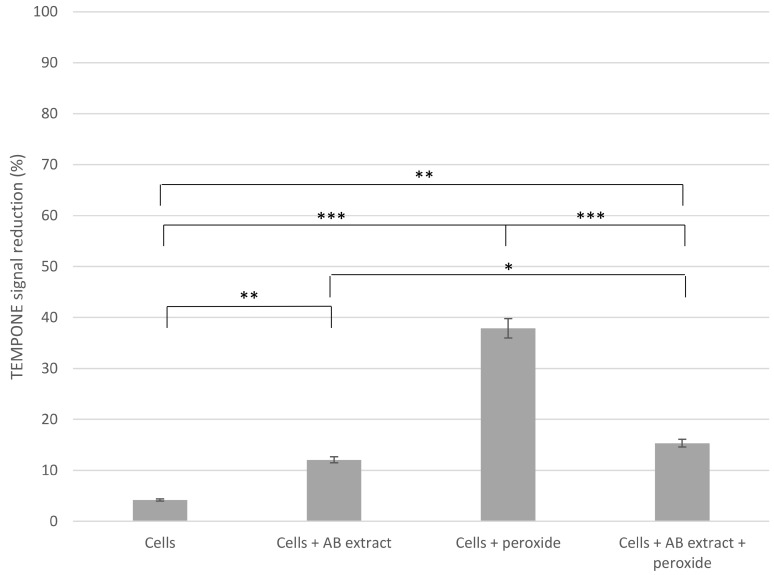
Effect of *A. bisporus* extract (AB) on TEMPONE oxidation in AmE-711 honey bee cells under basal and oxidative stress conditions. Data are presented as mean ± SE (*n* = 3 independent experiments). Significance brackets (independent *t*-test, unadjusted *p*-value): * *p* < 0.05, ** *p* < 0.01, *** *p* < 0.001. Statistical comparison was performed using one-way ANOVA followed by Tukey’s post hoc test.

In the case of untreated cell suspensions (Figure 2), the reduction in spin-probe signal intensity was minimal (4.20%), indicating a stable intracellular redox environment under basal conditions. The treatment of cells with the *A. bisporus* extract alone resulted in a moderate decrease in spin-probe signal (12.08%). Unlike highly phenolic-rich plant extracts [83,84,85], this response is more consistent with mild redox modulation rather than strong radical scavenging, suggesting that the mushroom extract may influence cellular redox status through metabolic or signaling-related mechanisms rather than acting as a potent ROS quencher.

The induction of oxidative stress through hydrogen peroxide treatment resulted in a significant TEMPONE signal reduction (37.88%). This confirms a strong peroxide-mediated oxidative response. Co-treatment with the *A. bisporus* extract significantly reduced this effect, lowering the TEMPONE signal to 15.34%. Importantly, this level approached that observed for cells treated with the extract alone, indicating that the extract was capable of buffering oxidative stress toward a controlled redox state rather than completely suppressing redox activity.

The antioxidant protective activity of *A. bisporus* extracts may not be exclusively associated with high concentrations of classical phenolic antioxidants, as is the case with plant extracts rich in polyphenols, such as grape pomace [86,87]. Based on our current results, we suggest that the biological activity of the extract may be attributable, at least in part, to the combined activity of low-molecular-weight metabolites, including mannitol, organic acids, amino acids, nucleotide derivatives, and phenolic constituents, which is consistent with literature data [88,89]. Many of these compounds are known to have a positive effect on cellular metabolism, stabilization of redox cofactors, or to indirectly enhance endogenous antioxidant systems rather than directly scavenging ROS [89]. This hypothesis, however, requires further investigation with quantitative metabolite analysis and targeted functional assays to establish causal relationships.

Another finding of our study related to the *A. bisporus* response, which was not appreciated enough, is the absence of excessive redox suppression under non-stressed conditions The moderate reduction in TEMPONE signal observed with extract treatment alone suggests that the extract does not induce reductive stress, a phenomenon increasingly recognized as a potential problem with high-dose antioxidant supplementation [9,90]. This balanced redox behavior we observed may be particularly desirable for honey bee cells, where tightly regulated ROS signaling is essential for immune responses and cellular homeostasis. Control experiments performed in culture medium with or without hydrogen peroxide, in the absence of the cells, showed no significant changes in TEMPONE signal, confirming that the observed effects were cell-dependent and biologically mediated.

From our results, it is evident that the *A bisporus* aqueous extract functions as a redox stabilizer rather than a strong antioxidant, since it is effective against peroxide-induced oxidative stress while preserving basal redox activity. This mode of action could be a more physiologically compatible strategy for mitigating oxidative stress in honey bees, as it differs from that of polyphenol-rich plant extracts. Since plant extracts are more potent free radical scavengers, they could cause strong suppression of ROS, which are, at the same time, essential for cell signaling [91]. Oxidative stress in honey bees is often increased, either by practices common for commercial beekeeping [92], such as colony migrations [93] or by acaricides used against honey bee mites [94], agropesticides and/or honey bee pathogens [3,33,95,96,97,98] or their combination [99], but also by food scarcity [93]. Furthermore, there are seasonal variations in redox homeostasis of honey bee workers, with an increase in oxidative stress at the end of winter [100,101], so we might hypothesize that *A. bisporus* extract, being capable of modulating redox processes, may benefit honey bees if administered in autumn, during the preparation of bee colonies for wintering.

Nevertheless, it should be noted that the AmE-711 cell line originates from honey bee embryos [102], so it is necessary to study the effects of *A. bisporus* extract in adult bees under laboratory and field conditions to validate the relevance of these findings and to assess the potential of our *A. bisporus* extract as a dietary supplement in apicultural practice.

## 3. Materials and Methods

### 3.1. Preparation of A. bisporus Water Extract

Extraction of bioactive compounds from commercially grown white button mushrooms (*A. bisporus*, strain A15) was performed using the method described in our previous study [33]. Distilled water was used to extract the finely powdered dried mushroom fruiting bodies for 60 min at 1.2 bar and 121 °C. Following extraction, the insoluble material was removed by filtration from the suspension, and the filtrate was concentrated to a third of its initial volume using a rotary evaporator. To precipitate the concentrated extract, 96% (*v*/*v*) ethanol was added, followed by incubation at 4 °C overnight. Centrifugation was used to gather the precipitate, which was subsequently dried at 40 °C and ground into a fine powder. The dried extract was stored at 4 °C until further use.

### 3.2. LC–MS Qualitative Analysis of Phenolic Compounds

Qualitative profiling of phenolic compounds present in the *A. bisporus* aqueous extract was performed using liquid chromatography–mass spectrometry (LC–MS), following the similar analytical protocol we have previously described [19]. Analyses were conducted using a Vanquish™ Core HPLC system (Thermo Fisher Scientific, Bremen, Germany) coupled to an Orbitrap Exploris 120 mass spectrometer equipped with a heated electrospray ionization (HESI) source (Thermo Fisher Scientific, Bremen, Germany). Chromatographic separation was achieved on a Syncronis C18 analytical column (100 × 2.1 mm, 1.7 µm particle size; Thermo Fisher Scientific, Bremen, Germany). The injection volume was 5 μL and the flow rate was constant at 300 μL/min. The compounds of interest were eluted with ultrapure water +0.1% formic acid (A) and acetonitrile (MS grade) + 0.1% formic acid (B): 5% B in the first min; 5–95% B from 1 to 10 min; 95% B from 10 to 12 min; 5% B until 15th min. The Orbitrap Exploris 120 mass spectrometer was equipped with an ESI source operating in negative ionization mode. Full scan MS was monitored from 100 to 1500 *m*/*z*, while data-dependent MS2 experiments were conducted with normalized collision energy of CID set to 35%. The dynamic exclusion time was set to 10 s with exclusion from a specific scan after 2 occurrences, and intensity threshold was set to 1 × 10^5^ [103]. Tentative identification of compounds was based on accurate monoisotopic mass measurements and MS^2^ fragmentation patterns in high resolution. Theoretical exact masses were calculated using ChemDraw software (v12.0, CambridgeSoft, Cambridge, MA, USA), while Xcalibur software (v2.1, Thermo Fisher Scientific, Waltham, MA, USA) was used for instrument control, data acquisition, and qualitative data analysis.

### 3.3. Honey Bee Cell Culture and Viability Assessment

The continuous honey bee cell line AmE-711, initially established by Goblirsch et al. [102], used in this study was provided by the Tick Cell Biobank, University of Liverpool (Liverpool, UK). The cytotoxicity of the *A. bisporus* aqueous extract toward honey bee cells was evaluated using the trypan blue exclusion assay, following the methodology of Phillips [104]; 25 µL of cell suspension (1 × 10^6^ cells/mL) was mixed with an equal volume of mushroom extract prepared at a concentration of 10 mg/mL (final concentration of extract was therefore 5 mg/mL). Cells in the negative control group were treated with phosphate-buffered saline (PBS) only, while cells in the positive control group were exposed to 100 µM hydrogen peroxide (H_2_O_2_). Samples were incubated for 1 h at 32 °C. Afterward, 25 µL of the cell suspension was mixed with 250 µL of 0.4% trypan blue solution and 725 µL of PBS. After 5 min, viable (unstained) and non-viable (stained) cells were counted using a Neubauer hemocytometer. All measurements were performed in triplicate. This cell line was originally derived from *Apis mellifera* embryonic tissue [102]. The final concentration of the extract in the assay was 5 mg/mL (i.e., equal volumes of cell suspension and 10 mg/mL extract solution were mixed 1:1). The final concentration of H_2_O_2_ in the positive control was 100 µM, prepared by dilution from a 10 mM stock in sterile deionized water.

### 3.4. Redox Response of Apis mellifera Cells to A. bisporus Extract Treatment

The redox activity of *Apis mellifera* AmE-711 cells (derived from honey bee embryonic tissues) treated with *A. bisporus* aqueous extract was studied using EPR spectroscopy, following the previously developed methodology [19,105]. Cells were cultured in Schneider’s Insect Medium and adjusted to a final concentration of 100,000 cells per sample in 27 µL of medium. All experimental samples had a final volume of 30 µL. Spin probe TEMPONE (2,2,6,6-tetramethyl-4-oxo-1-piperidinyloxy) was used at a final concentration of 0.025 mM, added as 1 µL of a 0.75 mM solution [105]. The mushroom extract was prepared as a 10 mg/mL solution in sterile deionized water. Oxidative stress was induced by adding 1 µL of 20 mM H_2_O_2_ (final concentration 0.67 mM). All solutions were prepared using sterile, deionized water.

Samples were prepared as follows and recorded two minutes after the addition of TEMPONE:

A. Controls

Water control: 29 µL deionized water + 1 µL TEMPONE.

Medium control: 27 µL Schneider’s Insect Medium + 2 µL deionized water + 1 µL TEMPONE.

Cell baseline: 27 µL cell suspension + 2 µL deionized water + 1 µL TEMPONE.

B. Mushroom Extract Effects

Extract in water: 28 µL deionized water + 1 µL extract + 1 µL TEMPONE.

Extract in medium: 27 µL Schneider’s Insect Medium + 1 µL extract + 1 µL deionized water + 1 µL TEMPONE.

Extract with cells: 27 µL cell suspension + 1 µL extract + 1 µL deionized water + 1 µL TEMPONE.

C. Cells under Oxidative Stress

Cells + H_2_O_2_: 27 µL cell suspension + 1 µL H_2_O_2_ + 1 µL deionized water + 1 µL TEMPONE.

Cells + extract + H_2_O_2_: 27 µL cell suspension + 1 µL extract + 1 µL H_2_O_2_ + 1 µL TEMPONE.

EPR spectra were recorded using a Bruker ELEXSYS II spectrometer (Bruker, Rheinstetten, Germany) operating in X-band at a microwave frequency of 9.8 GHz. Acquisition parameters were as follows [106]: microwave power 10 mW, modulation frequency 100 kHz, and modulation amplitude 0.2 mT.

### 3.5. Statistical Analysis

All experiments were conducted in triplicate, and results are presented as mean values ± standard error (SE). Statistical analysis was performed using Statistica 12.0 (64-bit) Statistical Software (StatSoft, Hamburg, Germany). Differences were considered statistically significant at *p* < 0.05. One-way ANOVA was used to compare normally distributed data among multiple groups, followed by Tukey’s HSD post hoc test where applicable. Normality was assessed using the Shapiro–Wilk test, and homogeneity of variances was verified using the Levene test.

## 4. Conclusions

An aqueous extract from the edible mushroom species *A. bisporus* has been shown in this study to have a balanced and biologically compatible redox-modulating activity on honey bee cells. High-resolution LC-MS analysis revealed that the extract is primarily composed of primary metabolites, including polyols, organic acids, amino acids, phosphorylated sugars, and nucleotide derivatives, with minor but biologically significant phenolic and lipid-related compounds, which together provide a plausible biochemical foundation for the observed cellular responses. The cell viability tests showed that the extract exhibited no significant cytotoxicity under the experimental conditions used. The results of EPR spectroscopy tests revealed that the extract did not cause excessive redox suppression under basal conditions. Under conditions of hydrogen peroxide-induced oxidative stress, the extract effectively attenuated redox imbalance and restored intracellular redox activity toward a controlled physiological range. In fact, *A. bisporus* extract is unlikely to exert its protective effects through high concentrations of classical antioxidants, but rather through the collective and synergistic actions of low-molecular-weight metabolites that support cellular metabolism, stabilize redox cofactors, and indirectly enhance endogenous antioxidant and stress-response systems.

The potential advantages of *A. bisporus* extracts for the entire organism, immune function, and resistance to environmental stressors such as pathogens and agrochemicals should be considered when analyzing the effects of dietary supplementation with these extracts. The strategy presented in this paper has the potential to be helpful in creating long-term dietary plans that promote the health of honey bees in contemporary apiculture.

At the end, it should be noted that this study was carried out on the AmE-711 cell line, derived from embryonic tissues [102], so further studies in adult bees and under field conditions are needed.

## Figures and Tables

**Table 1:** LC-MS data on metabolites identified in *Agaricus bisporus* extract.

No	Compound Name	*t*_R_, min	Molecular Formula, [M–H]^−^	Calculated Mass, *m*/*z*	Exact Mass, *m*/*z*	Δ mDa	MS^2^ Fragments, (% Base Peak)
1	Hexane-1,2,3,4,5,6-hexol	0.50	C_6_H_13_O_6_^−^	181.07189	181.07246	−0.57	59.01405(81), 71.01412(56), 85.02985(18), 89.02474(100), 101.02481(77), 181.07245(60)
2	Aspartic acid	0.51	C_4_H_6_NO_4_^−^	132.03020	132.03077	−0.57	88.04073(86), 95.02545(18), 113.03613(81), 114.02015(100), 132.03076(57)
3	Tetrose	0.52	C_4_H_7_O_4_^−^	119.03502	119.03563	−0.61	59.01407(93), 71.01427(35), 74.02512(78), 75.02862(62), 101.02441(21), 119.03564(100)
4	Deoxyhexose	0.52	C_6_H_11_O_5_^−^	163.06120	163.06192	−0.72	59.01406(73), 85.02994(53), 101.02490(34), 113.02499(10), 131.03543(9), 163.0619(100)
5	Hexose	0.52	C_6_H_11_O_6_^−^	179.05619	179.05685	−0.66	89.02477(81), 99.00914(35), 131.03560(31), 161.04614(65), 179.05685(100)
6	Glutamic acid	0.53	C_5_H_8_NO_4_^−^	146.04596	146.04650	−0.54	102.05645(100), 128.03589(54), 146.04649(43)
7	Threonine	0.53	C_4_H_8_NO_3_^−^	118.05100	118.05142	−0.42	72.00938(4), 74.02502(100), 118.05149(29)
8	Tetrahydroxypentanoic acid	0.54	C_5_H_9_O_6_^−^	165.04046	165.04100	−0.54	59.01407(15), 75.00906(100), 99.00917(12), 129.0199(14), 147.03053(9), 165.04111(61)
9	Glucose phosphate	0.55	C_6_H_12_O_9_P^−^	259.02247	259.02302	−0.55	78.95934(78), 96.96997(100), 138.98085(13), 241.01292(6), 259.02301(10)
10	Malic acid	0.55	C_4_H_5_O_5_^−^	133.01420	133.01483	−0.63	71.01413(38), 89.02479(7), 115.00418(100), 133.01483(47)
11	Fumaric acid	0.56	C_4_H_3_O_4_^−^	115.00370	115.00419	−0.49	71.01411(100), 115.00419(17)
12	*N*-(1-Deoxy-D-mannitol-1-yl)-L-glutamic acid	0.56	C_11_H_20_NO_9_^−^	310.11445	310.11540	−0.95	128.03580(100)
13	Pyroglutamic acid	0.57	C_5_H_6_NO_3_^−^	128.03532	128.03584	−0.52	128.03583(100)
14	Glycerol phosphate	0.57	C_3_H_8_O_6_P^−^	171.00646	171.00712	−0.66	78.95934(100), 96.96999(15), 171.00711(15)
15	*N*-Acetylglucosamine phosphate	0.58	C_8_H_15_NO_9_P^−^	300.04909	300.04990	−0.81	78.95931(100), 96.96993(82), 118.05138(33), 138.98077(5), 199.00186(4), 300.04990(6)
16	Uridine diphosphate *N*-acetylglucosamine (UDP-GlcNAc)	0.58	C_17_H_26_N_3_O_17_P_2_^−^	606.07435	606.07622	−1.87	78.95933(93), 96.96993(33), 158.92595(71), 176.93655(37), 272.95801(100), 282.03943(73), 362.00555(20), 384.98541(91), 402.99573(20)
17	Methylcitric acid	0.59	C_7_H_9_O_7_^−^	205.03538	205.03575	−0.38	71.05051(14), 87.00906(28), 99.04551(26), 101.02478(26), 125.02486(100), 145.01480(20)
18	Uridine monophosphate (5′-UMP)	0.60	C_9_H_12_N_2_O_9_P^−^	323.02866	323.02895	−0.29	78.95933(100), 96.96996(84), 111.02045(18), 211.00206(15)
19	Aconitic acid	0.60	C_6_H_5_O_6_^−^	173.00916	173.00975	−0.59	85.02982(42), 111.00922(100), 129.01987(9)
20	2-Furoic acid	0.60	C_5_H_3_O_3_^−^	111.00880	111.00938	−0.58	67.01921(49), 111.00937(100)
21	Citric acid	0.60	C_6_H_7_O_7_	191.01973	191.02040	−0.67	85.02983(34), 87.00908(50), 111.00921(100), 129.01985(8), 173.00999(2), 191.02039(8)
22	Succinic acid	0.67	C_4_H_5_O_4_^−^	117.01930	117.01985	−0.55	73.02979(100), 99.0092(10), 117.01984(41)
23	Azelaic acid	7.07	C_9_H1_5_O_4_^−^	187.09758	187.09832	−0.73	97.06628(5), 125.09771(100), 169.08774(4), 187.0983(41)
24	Phelligridin D	7.83	C_20_H_11_O_8_^−^	379.04592	379.04736	−1.44	229.01508(16), 269.01013(100), 307.06235(17), 335.05756(36), 351.05225(16), 379.04736(70)
25	Phelligridin C	8.21	C_20_H_11_O_7_^−^	363.05108	363.05220	−1.12	217.01526(22), 269.01038(19), 307.06207(17), 319.06281(21), 335.05804(17), 363.05225(100)
26	Dihydroxyoctadecadienoic acid	8.84	C_18_H_31_O_4_^−^	311.22285	311.22495	−2.10	249.22385(8), 293.21329(100), 311.22495(41)
27	Betulin-3-caffeate	11.89	C_39_H_55_O_5_^−^	603.40552	603.40724	−1.72	161.02512(2), 603.40723(100)
28	Hydroxytetracosanoic acid	12.44	C_24_H_47_O_3_^−^	383.35307	383.35415	−1.08	337.34888(65), 365.34149(2), 383.35443(100)
29	Hydroxydocosanoic acid	13.88	C_22_H_43_O_3_^−^	355.32177	355.32278	−1.01	309.31738(64), 337.31339(2), 355.32278(100)

## Data Availability

All data underlying the results are available as part of the article and no additional source data are required.

## Supplementary Materials

The following supporting information can be downloaded at: https://www.mdpi.com/article/10.3390/molecules31122011/s1, Figure S1: Cell viability (%) of AmE-711 honey bee cells in the negative control (PBS), *A. bisporus* extract-treated (5 mg/mL), and positive control (100 µM H_2_O_2_) groups. Data are expressed as mean ± SE (*n* = 3). One-way ANOVA revealed no statistically significant differences (n.s.) among groups (F(2,6) = 2.58, *p* = 0.155).
