# Supplementary material for: Metabolic Redox Modulation by Agaricus bisporus Aqueous Extract in Honey Bee Cells
Source: Molecules. 2026 Jun 9;31(12):2011. doi: 10.3390/molecules31122011 (PMC13304580; doi:10.3390/molecules31122011)
Supplement: Supplementary file 1 [file molecules-31-02011-s001.zip › molecules-4343718-supplementary.pdf]

Supplementary File

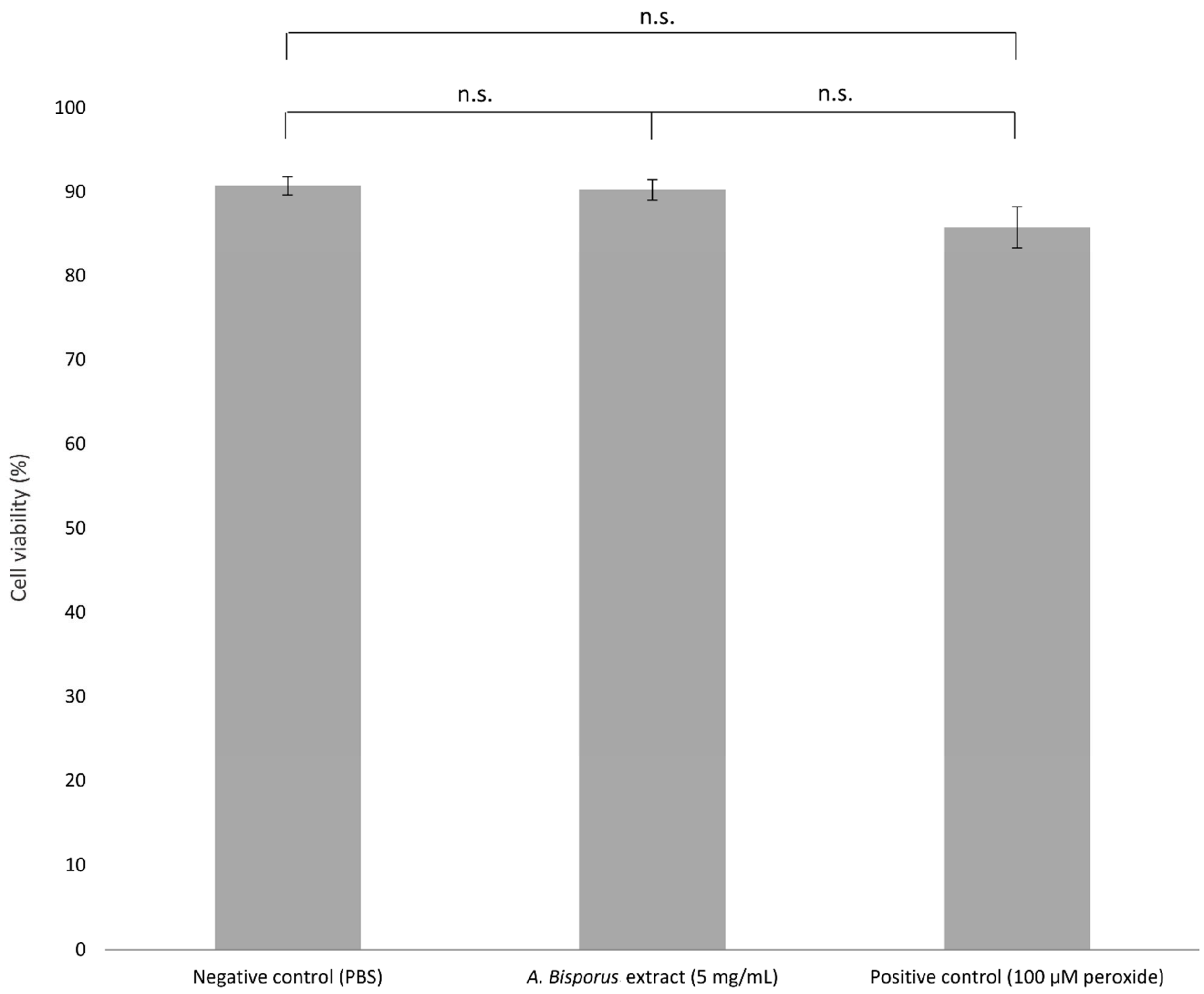

Figure S1. Cell viability (%) of AmE-711 honey bee cells in the negative control (PBS), *A. bisporus* extract-treated (5 mg/mL), and positive control (100 μM H_2O_2) groups. Data are expressed as mean ± SE ($n = 3$). One-way ANOVA revealed no statistically significant differences (n.s.) among groups ($F(2,6) = 2.58$, $p = 0.155$).
